# Development, Validation, and Clinical Utility Assessment of a Prognostic Score for 1-Year Unplanned Rehospitalization or Death of Adult Sepsis Survivors

**DOI:** 10.1001/jamanetworkopen.2020.13580

**Published:** 2020-09-14

**Authors:** Manu Shankar-Hari, Gordon D. Rubenfeld, Paloma Ferrando-Vivas, David A. Harrison, Kathryn Rowan

**Affiliations:** 1 Guy’s and St Thomas’ NHS Foundation Trust, ICU Support Offices, St Thomas’ Hospital, London, United Kingdom; 2 School of Immunology & Microbial Sciences, King’s College London, London, United Kingdom; 3 Interdepartmental Division of Critical Care Medicine, Sunnybrook Health Sciences Centre, Toronto, Ontario, Canada; 4 Associate Editor, *JAMA Network Open*; 5 Intensive Care National Audit & Research Centre, Napier House, London, United Kingdom

## Abstract

**Importance:**

The longer-term risk of rehospitalizations and death of adult sepsis survivors is associated with index sepsis illness characteristics.

**Objective:**

To derive and validate a parsimonious prognostic score for unplanned rehospitalizations or death in the first year after hospital discharge of adult sepsis survivors.

**Design, Setting, and Participants:**

This cohort study used data from the Intensive Care National Audit & Research Centre Case Mix Programme database on adult sepsis survivors identified from consecutive critical care admissions to 192 adult general critical care units in England, United Kingdom, between April 1, 2009, and March 31, 2014 (94 748 patients in the derivation cohort), and between April 1, 2014, and March 31, 2015 (24 669 patients in the validation cohort). Statistical analysis was performed from July 5 to October 31, 2019. Generic characteristics (age, sex, race/ethnicity, 2015 Index of Multiple Deprivation [IMD2015] in England quintiles, preadmission dependence, previous hospitalizations in the year preceding index sepsis admission, comorbidity, admission type, Acute Physiology and Chronic Health Evaluation II physiology score, hospital length of stay, worst blood lactate and blood hemoglobin concentrations, and type of hospital) and sepsis-specific characteristics (site of infection, numbers of organ dysfunctions, and organ support) at the index sepsis admission were used as predictors.

**Main Outcomes and Measures:**

Prognostic score derived and validated using multivariable logistic regression for the outcome of unplanned rehospitalization or death in the first year after hospital discharge of adult sepsis survivors, as well as clinical usefulness assessed using decision curve analysis. Prognostic score validation was performed for internal validation with bootstrapping and temporal cohort external validation.

**Results:**

This cohort study included 94 748 patients (51 164 men [54.0%]; mean [SD] age, 61.3 [17.0] years) in the derivation cohort and 24 669 patients (13 255 men [53.7%]; mean [SD] age, 62.1 [16.8%]) in the validation cohort. Unplanned rehospitalization or death in the first year after hospital discharge occurred for 48 594 patients (51.3%) in the derivation cohort and 13 129 patients (53.2%) in the validation cohort. Eight independent predictors were identified and weighted to generate a prognostic score for every patient: previous hospitalizations, age in 10-year increments, IMD2015 in England quintiles, preadmission dependence, comorbidities, admission type, blood hemoglobin level, and site of infection. The total prognostic score ranged from 0 to 22 points, with lower scores indicating a lower risk of the outcome. The derivation and validation cohorts had similar rates of prognostic scores of 0 to 4 points (5088 of 16 684 patients [30.5%] and 471 of 1725 patients [27.3%]) and prognostic scores of 11 points or more (15 732 of 21 641 patients [72.7%] and 5753 of 7952 patients [72.3%]). The area under the receiver operating characteristic curve for the prognostic score was 0.675 (95% CI, 0.672-0.679). The decision curve analysis highlighted an optimal score cutoff of 7 points or more.

**Conclusions and Relevance:**

The prognostic score reported in this study uses 8 internationally feasible predictors measured during the index sepsis admission and provides clinically useful information on sepsis survivors’ risk of unplanned rehospitalization or death in the first year after hospital discharge.

## Key Points

**Question:**

Could a prognostic score for unplanned rehospitalizations or death in the first year after hospital discharge of adult sepsis survivors be developed using index sepsis illness characteristics as predictors?

**Findings:**

In this cohort study of 94 748 patients in adult general critical care units in England, unplanned rehospitalization or death in the first year after hospital discharge occurred for 51% of patients in the derivation cohort and 53% of patients in the validation cohort. The prognostic score is calculated using 8 predictors: previous hospitalizations in the preceding year, age, socioeconomic status, preadmission dependence, number of comorbidities, admission type, site of infection, and admission blood hemoglobin level.

**Meaning:**

This score provides clinically useful information for prognosis discussions and planning follow-up care for sepsis survivors.

## Teaser

This cohort study uses data from the Intensive Care National Audit & Research Centre Case Mix Programme database to derive and validate a parsimonious prognostic score for unplanned rehospitalizations or death in the first year after hospital discharge of adult sepsis survivors.

## Introduction

Sepsis is defined as life-threatening organ dysfunction caused by a dysregulated host response to infection.^1^ Studies, mainly from critical care settings in high-income countries, consistently report an increasing incidence of sepsis, as well as improving acute mortality from sepsis.^2,3,4^ This epidemiologic pattern is associated with increasing numbers of patients surviving to hospital discharge after a critical care unit admission for sepsis, who are hereafter referred to as *sepsis survivors*. Sepsis survivors have ongoing health care needs and are at increased risk of adverse outcomes.^5^

Two common and patient-centered adverse outcomes for sepsis survivors are the greater risk of rehospitalization and of long-term mortality compared with survivors of nonsepsis hospitalizations. Systematic reviews have reported a mean 1-year rehospitalization rate of 39.0% (95% CI, 22.0%-59.4%)^6^ and a mean 1-year mortality rate of 16.1% (95% CI, 14.1%-18.1%) among adult sepsis survivors.^7^ A systematic review of rehospitalization risk factors (predictors) for sepsis survivors that included 56 studies highlighted generic characteristics (such as older age, male sex, comorbidities, nonelective admissions, and hospitalizations prior to index sepsis admission) and index sepsis admission characteristics (such as infection and illness severity) as consistent predictors across studies.^6^ The long-term mortality risk for sepsis survivors is also associated with these acute illness characteristics.^8^ More important, because health care professionals will know these acute illness characteristics before the sepsis survivors are discharged, it is feasible to use these characteristics to predict the risk of these patient-centered adverse outcomes.

With the use of these acute illness characteristics, there are no prognostic scores to assess the 1-year risk of unplanned rehospitalizations or death after hospital discharge for adult sepsis survivors. Such a score might provide clinicians with objective prognostic information to supplement their clinical judgment when advising patients and families, to help identify a higher-risk sepsis survivor population for targeted follow-up care, and to design trials aiming at improving sepsis survivor outcomes.^9,10,11^ Therefore, we derived and validated a parsimonious prognostic score for the outcome of unplanned rehospitalization or death in the first year after hospital discharge of adult sepsis survivors, using multivariable logistic regression.^12^ We then assessed the clinical usefulness of the prognostic score using decision curve analysis (DCA),^13^ which informs clinicians when to intervene selectively^13^ and, more importantly, where data on treatment effectiveness and patient preferences do not exist,^14^ which is the current scenario for sepsis survivors.

## Methods

### Study Database and Approvals

Data were extracted from the Intensive Care National Audit & Research Centre Case Mix Programme database,^15^ which is the national clinical audit covering all adult critical care units in England, United Kingdom, collecting data on consecutive critical care admissions. This anonymized study data set was linked with the Hospital Episode Statistics database and Office for National Statistics death registrations,^16^ using patient-level unique identifiers.^15^ Approval for the collection and use of these data has been obtained under Section 251 of the National Health Service Act 2006. Waiver of informed consent for use of this data was obtained from Wales Research Ethics Committee 5 and Confidentiality Advisory Group, which approved this study as part of the Long-term Outcomes After Critical Illness Project. Our study conforms to the Transparent Reporting of a Multivariable Prediction Model for Individual Prognosis or Diagnosis (TRIPOD) reporting guideline^12^ for deriving and validating prognostic scores (eFigure 1 in the Supplement).

### Patient Population and Outcome Assessment

From consecutive critical care admissions to 192 of 200 adult general critical care units (96%) in England, we identified a cohort of sepsis survivors, defined as adult patients who survived to hospital discharge after an index critical care admission for sepsis meeting Sepsis-3 criteria.^1^ We have previously reported 5-year mortality outcomes in this sepsis survivor cohort.^8^ Unplanned rehospitalization episodes and rehospitalization diagnoses were ascertained from the linked data set and grouped as per the Healthcare Cost and Utilization Project Clinical Classifications Software multilevel categories.^17^ This mapping was similar to previous reports in the literature.^18,19^

### Derivation and Validation Cohorts

The derivation cohort included all consecutive admissions between April 1, 2009, and March 31, 2014, with the date of rehospitalization or death ascertained as of March 31, 2015.^8^ The validation cohort included all consecutive admissions between April 1, 2014, and March 31, 2015, with the date of rehospitalization or death ascertained as of March 31, 2016. This validation cohort meets the temporal (narrow external) validation definitions in the TRIPOD statement^12^ because it uses the same predictors, the same outcome definitions, and the same measurements from the same setting, but sampled from a later period.

### Statistical Analysis

#### Identification and Handling of Predictors in All Models

Statistical analysis was performed from July 5 to October 31, 2019. Potential generic predictors and sepsis-specific predictors^8^ associated with either all-cause rehospitalization or 1-year mortality among sepsis survivors were identified from systematic reviews^6,7^ (eTable 1 in the Supplement). The generic predictors were as follows: the Acute Physiology and Chronic Health Evaluation (APACHE) II physiology score^20^ in 5-point increments, the hospital length of stay in 7-day increments, the number of hospitalizations in the year preceding the index sepsis admission (base category = none), age in 10-year increments (base category = 18-30 years), race/ethnicity (base category = White), quintiles of 2015 Index of Multiple Deprivation (IMD2015) in England as a proxy for socioeconomic status (base category = least deprived), prehospitalization dependence status (base category = no dependence), the number of comorbidities (base category = none), surgical status (base category = medical), admission blood hemoglobin level (≤7, 7.1-11, and 9.1-11 g/dL, with a base category of >11 g/dL [to convert to grams per liter, multiply by 10.0]), and admission blood lactate concentrations (18-36 mg/dL and >36 mg/dL, with a base category of <18 mg/dL [to convert to millimoles per liter, multiply by 0.111]), with sex (base category = female) and hospital type (base category = nonuniversity) as binary predictors. The sepsis-specific predictors were as follows: site of infection (respiratory = base category) and numbers of organ dysfunction^1,3^ (base category = 1 organ dysfunction), with types of organ support (renal, respiratory, gastrointestinal, neurologic, and cardiovascular) at any point during the critical care unit stay^21^ as binary predictors.

The blood hemoglobin level cutoffs were chosen based on the transfusion guidelines for critically ill patients (transfusion threshold, <7 g/dL; target level, 7-9 g/dL)^22^ and blood lactate level cutoffs were based on the literature.^23^ The most severe comorbidity was identified for each organ system; individual comorbidities were given equal weights. Further details on predictors are provided in the eMethods in the Supplement.

#### Prognostic Model Development

The prognostic model was derived using multivariable logistic regression with unplanned rehospitalization or death in the first year after hospital discharge as the outcome. The prognostic model derivation occurred in the following steps, after all the described predictors underwent univariable testing for association with the outcome. First, the preliminary model with all the predictors was generated. Second, the post–stepwise reduction model was generated with the stepwise reduction method used to remove the least-significant predictors, until all remaining predictors had a significance of *P* < .001. Third, because our goal was to derive a parsimonious prognostic score, we sequentially eliminated complex predictors and predictors with the lowest coefficients to generate the final model with the final set of predictors. At each step, the discrimination of the model was assessed by the C statistic (area under the receiver operating characteristic curve [AUROC]) and the overall accuracy was assessed by the Brier score. Multicollinearity was assessed with variance inflation factors^24^ for both the preliminary model with all predictors and the final model.

#### Prognostic Score Development

We assigned the largest coefficient in the final model 5 points and rescaled all other coefficients relative to this point value, rounding to the nearest integer. We then calculated a score for each patient by totaling the points based on individual predictors and assessed the distribution. The derivation cohort was then stratified on outcome risk based on the individual scores. The predictive accuracy of the score was then assessed by comparing predicted vs observed outcome (calibration) and discriminant validity with AUROC.^25^

#### Assessment of Clinical Utility of Prognostic Score

Sepsis survivors and clinicians decide together on follow-up care, based on patients’ risk and benefit preferences. Clinicians can either provide follow-up care to all sepsis survivors (treat all) or provide no follow-up care (treat none) or use the prognostic information assessed with DCA to intervene selectively. Decision curve analysis is underpinned by 2 concepts: threshold probability and net benefit. Threshold probability of outcome refers to the empirical value above which a clinician would choose to treat or a sepsis survivor would choose to receive treatment. In other words, the sepsis survivors make a conscious decision that the benefit of their empirical treatment outweighs the harms associated with the treatment. The net benefit is the difference between the expected benefit and the expected harm. The expected benefit here is that sepsis survivors who will experience 1 or more rehospitalizations or death within 1 year after hospital discharge will choose to receive treatment (true positive). The expected harm is that sepsis survivors who will not experience this outcome will choose to receive treatment (false positive) multiplied by those sepsis survivors’ threshold probabilities. Decision curve analysis uses this theoretical association of net benefit = true positive rate − [false positive rate × [threshold probability/(1 − threshold probability)] to generate decision curves.^14^ The highest decision curve over the full range of threshold probabilities is the best cutoff for a prognostic score, regardless of sepsis survivors’ preferences. However, if the decision curves cross, then the optimal cutoff will be associated with sepsis survivors’ preferences of threshold probability.

#### Prognostic Score Validation

We performed internal validation with bootstrapping^12^ and temporal cohort external validation.^12^ Bootstrap samples (n = 200) were taken from the original data, stepwise selection repeated within each bootstrap sample, with Efron optimism estimated using the difference between the bootstrap data and the original data.^26^ Bootstrapping provides the nonparametric maximum likelihood of error and stable estimates with low risk of bias,^27^ assuming that the derivation cohort represents a random sample of sepsis survivors.

Reported *P* values are 2-sided, and *P* < .05 was considered to represent a statistically significant result. Continuous data were summarized as mean (SD) values, when normally distributed, and as median values and interquartile ranges, when not normally distributed. Categorical data were presented as frequency and percentage. We achieved 96% outcome data linkage.^8^ Because missing data were less than 0.5% for all the predictors and because the Case Mix Programme had between 90% and 100% coverage of adult general critical care units in England during the study period, we performed full case analyses, assuming that data are missing completely at random.^28^ We did not perform an a priori sample size calculation. All analyses were performed using Stata/SE, version 14.0 (StataCorp LLC).

## Results

### Derivation and Validation Cohorts

**Figure 1.**
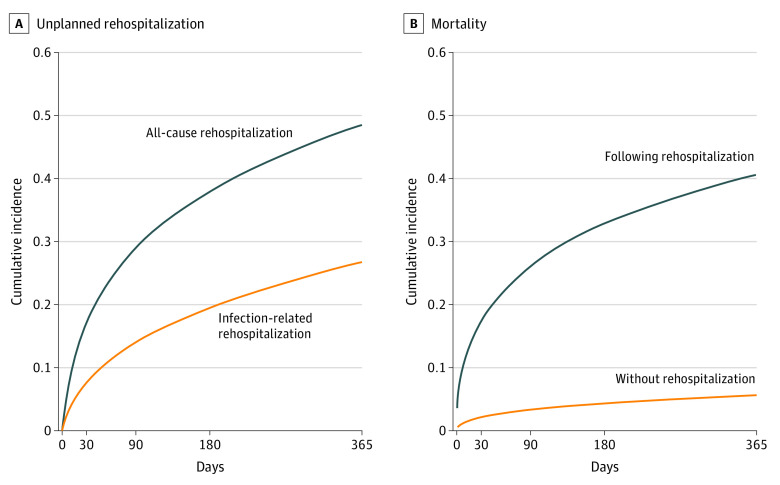
Cumulative Incidence Plots for Unplanned Rehospitalization, Infection-Related Rehospitalization, and Mortality by Rehospitalization Status A, The cumulative all-cause unplanned rehospitalization rates (blue line) at 30, 90, 180, and 365 days were 16.9%, 28.4%, 36.9%, and 46.9%, respectively. The cumulative infection-related rehospitalization rates (orange line) at 30, 90, 180, and 365 days were 7.7%, 14.1%, 19.4%, and 26.7%, respectively (see eTable 3 in the Supplement for further information). B, The cumulative all-cause mortality rates among patients with 1 or more rehospitalizations (blue line) at 30, 90, 180, and 365 days were 17.4%, 26.2%, 32.8%, and 40.5%, respectively. These are reported using Cox proportional hazards regression models.

There were 94 748 sepsis survivors in the derivation cohort and 24 669 sepsis survivors in the validation cohort. The study outcome of unplanned rehospitalization or death in the first year after hospital discharge occurred for 48 594 patients (51.3%) in the derivation cohort and 13 129 patients (53.2%) in the validation cohort (Figure 1). The generic and sepsis-specific characteristics were similar between the derivation and validation cohorts (Table 1; eTable 2 in the Supplement).

**Table 1.** Clinical and Outcome Characteristics of the At-Risk Sepsis Survivors in the Derivation Cohort and the Validation Cohort

Parameter	Derivation cohort (n = 94 748)	Validation cohort (n = 24 669)
Age, mean (SD), y	61.3 (17.0)	62.1 (16.8)
Women, No. (%)	43 584 (46.0)	11 414 (46.3)
Race/ethnicity, No. (%)		
White	86 056 (90.8)	22 143 (89.7)
Asian	3378 (3.6)	968 (3.9)
Black	2020 (2.1)	576 (2.3)
Other^a^	3294 (3.5)	982 (4.0)
IMD2015 in England quintiles, No. (%)		
1 (Least deprived)	14 773 (15.6)	3737 (15.2)
2	16 442 (17.4)	4156 (16.9)
3	18 478 (19.5)	4732 (19.2)
4	20 152 (21.3)	5086 (20.6)
5 (Most deprived)	24 587 (26.0)	6006 (24.4)
Preadmission dependence, No. (%)		
None	70 737 (74.7)	17 925 (72.7)
Moderate (some assistance required with ADLs)	22 427 (23.7)	6300 (25.5)
Severe (total assistance required with ADLs)	1187 (1.3)	342 (1.4)
Comorbidities, No. (%)		
0	57 885 (61.1)	14 232 (57.7)
1	19 995 (21.1)	5131 (20.8)
2	10 640 (11.2)	3148 (12.8)
3	4450 (4.7)	1438 (5.8)
≥4	1778 (1.9)	720 (2.9)
Hospitalizations in preceding year, No. (%)		
0	41 691 (44.0)	10 429 (42.3)
1	20 717 (21.9)	5272 (21.4)
2	11 899 (12.6)	3082 (12.5)
≥3	20 441 (21.6)	5886 (23.9)
Admission type, No. (%)		
Medical	65 692 (69.3)	17 379 (70.5)
Elective surgical	5526 (5.8)	1345 (5.5)
Emergency surgical	23 521 (24.8)	5943 (24.1)
Index illness severity, mean (SD)		
APACHE II score	16.7 (5.9)	16.6 (7.4)
APACHE II physiology score	12.4 (5.2)	12.2 (5.2)
ICNARC physiology score	18.3 (7.4)	18.0 (7.4)
Site of infection, No. (%)		
Respiratory	43 858 (46.3)	11 348 (46.0)
Cardiovascular	1612 (1.7)	391 (1.6)
Gastrointestinal	28, 630 (30.2)	7210 (29.2)
Genitourinary	6747 (7.1)	1960 (8.0)
Musculoskeletal or dermatologic	5075 (5.4)	1430 (5.8)
Neurologic	3206 (3.4)	781 (3.2)
Unknown	5620 (5.9)	1549 (6.3)
Organ dysfunction, No. (%)		
1	9728 (10.3)	2584 (10.5)
2	26 878 (28.4)	7386 (29.9)
3	31 119 (32.8)	7883 (32.0)
4	21 075 (22.2)	5362 (21.7)
5	5949 (6.3)	1454 (5.9)
Organ support, No. (%)		
Advanced respiratory support	47 720 (50.4)	11 028 (44.7)
Advanced cardiovascular support	23 359 (24.7)	5421 (22.0)
Renal support	10 369 (10.9)	2490 (10.1)
Gastrointestinal support	44 973 (47.5)	10 583 (42.9)
Neurologic support	5527 (5.8)	1297 (5.3)
Hospital length of stay during index hospitalization, median (IQR), d	21 (11-40)	20 (11-37)
Type of hospital, No. (%)		
Nonuniversity	63 814 (67.4)	16 160 (65.5)
University or university affiliated	30 934 (32.7)	8509 (35.5)
All-cause rehospitalization or death by 365 d, No./total No. (%)	48 594/94 748 (52.2)	13 129/24 669 (53.2)
All-cause rehospitalizations by 365 d, No./total No. (%)	44 559/94 748 (47.0)	12 011/24 669 (48.7)
All-cause rehospitalization		
0	50 188/94 748 (53.0)	12 658/24 669 (51.3)
1	21 619/94 748 (22.8)	5631/24 669 (22.8)
2	10 331/94 748 (10.9)	2804/24 669 (11.4)
3	5367/94 748 (5.7)	1505/24 669 (6.1)
≥4	7243/94 748 (7.6)	2071/24 669 (8.4)
Time to first all-cause rehospitalization by 365 d, median (IQR), d	55 (16-153)	52 (14-158)
Death by 365 d, No./total No. (%)		
Overall	13 819/94 748 (14.6)	3712/24 669 (15.0)

Abbreviations: ADLs, activities of daily living; APACHE, Acute Physiology and Chronic Health Evaluation; ICNARC, Intensive Care National Audit & Research Centre; IMD2015, 2015 Index of Multiple Deprivation in England; IQR, interquartile range.

^a^ Other includes mixed, not stated, and any other ethnic group.

### Prognostic Model Development

On univariable testing, age, sex, race/ethnicity, IMD2015 in England quintiles, preadmission dependence, all comorbidities, any hospitalization in the preceding year, admission type, illness severity, hospital length of stay, lower blood hemoglobin level at index admission, site of infection, numbers of organ dysfunction, and organ support were significantly associated with the study outcome of unplanned rehospitalization or death in the first year after hospital discharge (eTable 4 in the Supplement).

The preliminary model with all predictors is shown in eTable 5 in the Supplement. The stepwise reduction procedure to generate the poststepwise reduction model removed the following predictors: sex, blood lactate level, numbers of organ dysfunction, and organ support (respiratory, renal, gastrointestinal, and neurologic). We then sequentially eliminated complex predictors (APACHE II acute physiology score) and predictors with the lowest coefficients (race/ethnicity, advanced cardiovascular support, hospital length of stay, and hospital type). This did not alter the discrimination and calibration of the model (eTable 5 in the Supplement). All variance inflation factors were considerably less than 10 (with a mean value of 2.2 for the preliminary model and of 2.4 for the final model). The predictors in the final model were previous hospitalization, age, IMD2015 in England quintiles, preadmission dependence, number of comorbidities, admission type, blood hemoglobin level at admission, and site of infection (Table 2).

**Table 2.** Predictors in the Final Model for Calculating the Prognostic Score in the Derivation Cohort^a^

Risk factor known at index sepsis admission	No. with outcome/No. at risk (%)	Adjusted odds ratio (95% CI)	*P* value^b^	Points
Previous hospitalizations				
0	16 761/41 691 (40.2)	1 [Reference]	<.001	0
1	10 458/20 717 (50.5)	1.22 (1.18-1.27)		1
2	6933/11 899 (58.3)	1.47 (1.40-1.54)		2
≥3	14 442/20 441 (70.7)	2.19 (2.09-2.30)		4
Age in 10-y increments				
<30	2233/5683 (39.3)	1 [Reference]	<.001	0
30-39	2578/6151 (41.9)	1.10 (1.01-1.18)		0
40-49	5173/10 731 (48.2)	1.35 (1.26-1.44)		1
50-59	7411/14 830 (50.0)	1.36 (1.27-1.45)		1
60-69	11 675/22 380 (52.2)	1.44 (1.35-1.53)		2
70-79	12 289/22 518 (54.6)	1.59 (1.49-1.69)		2
≥80	7235/12 455 (58.1)	1.94 (1.81-2.07)		3
IMD2015 quintile				
1 (Least deprived)	7233/14 773 (49.0)	1 [Reference]	<.001	0
2	8291/16 442 (50.4)	1.05 (1.00-1.09)		0
3	9392/18 478 (50.8)	1.07 (1.02-1.12)		0
4	10 363/21 152 (51.4)	1.10 (1.06-1.16)		0
5 (Most deprived)	13 224/24 587 (53.8)	1.23 (1.18-1.29)		1
Preadmission dependence				
None	33 254/70 737 (47.0)	1 [Reference]	<.001	0
Moderate	14 323/22 427 (63.9)	1.54 (1.49-1.59)		2
All	836/1187 (70.4)	2.80 (2.45-3.20)		5
Comorbidities				
0	24 413/57 885 (42.2)	1 [Reference]	<.001	0
1	11 974/19 995 (59.9)	1.39 (1.33-1.44)		2
2	7364/10 640 (69.2)	1.72 (1.63-1.82)		3
3	3384/4450 (76.0)	2.18 (2.02-2.36)		4
≥4	1459/1778 (82.1)	2.74 (2.41-3.12)		5
Admission				
Elective surgical	2595/5526 (47.0)	1 [Reference]	<.001	0
Emergency surgical	11 274/23 521 (47.9)	1.29 (1.21-1.37)		1
Medical	34 722/65 692 (52.9)	1.48 (1.39-1.57)		2
Blood hemoglobin level at admission				
>11	14 164/31 151 (45.5)	1 [Reference]	<.001	0
9.1-11	17.583/34 461 (51.0)	1.10 (1.06-1.14)		0
7.1-9	13 715/23 890 (57.4)	1.28 (1.23-1.33)		1
≤7	2590/4202 (61.6)	1.46 (1.36-1.56)		2
Site of infection				
Neurologic	1203/3206 (37.5)	1 [Reference]	<.001	0
Respiratory	22 928/43 858 (52.3)	1.30 (1.20-1.40)		1
Cardiovascular	939/1612 (58.3)	1.45 (1.27-1.65)		2
Gastrointestinal	14 175/28 630 (49.5)	1.30 (1.19-1.41)		1
Genitourinary	3582/6747 (53.1)	1.19 (1.08-1.30)		1
Musculoskeletal or dermatologic	2544/5075 (50.1)	1.17 (1.06-1.29)		0
Unknown	3223/5620 (57.4)	1.33 (1.21-1.46)		1
Score category				
0-4 Points (risk strata = 1)	5088/16 684 (30.5)	1 [Reference]	NA	NA
5-6 Points (risk strata = 2)	10 637/25 631 (41.5)	1.62 (1.55-1.68)		NA
7-10 Points (risk strata = 3)	17 137/30 791 (55.7)	2.68 (2.55-2.77)		NA
≥11 Points (risk strata = 4)	15 732/21 641 (72.7)	5.42 (5.20-5.65)		NA
Total	48 594/94 748 (51.3)	NA	NA	NA

Abbreviations: IMD2015, 2015 Index of Multiple Deprivation; NA, not applicable.

^a^ Predictors associated with all rehospitalizations or death in the first year after surviving hospitalization for sepsis-related critical illness included in the parsimonious prognostic score in the derivation cohort and validation cohort. The area under the receiver operating characteristic curve from the adjusted model was 0.684 (95% CI, 0.672-0.688) and Brier score was 0.23. The final integer score had an area under the receiver operating characteristic curve of 0.673 (95% CI, 0.670-0.677).

^b^ Representing the test for homogeneity in odds ratio for categories within groups, estimated using postestimation commands after the logistic regression model.

### Prognostic Score Development

**Figure 2.**
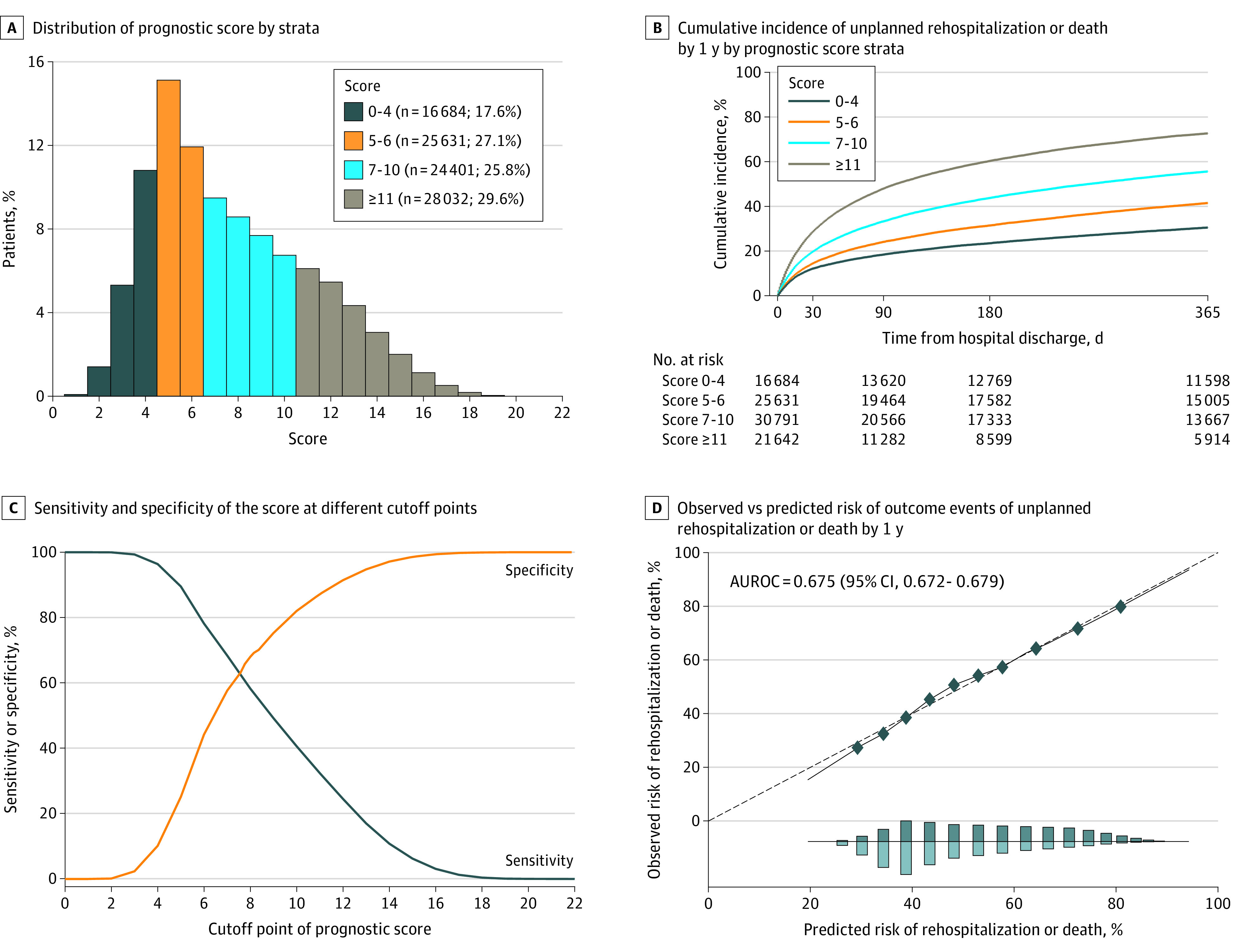
Description and Assessment of Clinical Prediction Model in Derivation Cohort A, Distribution of prognostic score by strata. B, Cumulative incidence of unplanned rehospitalization or death at 1 year after hospital discharge by prognostic score strata. C, Sensitivity and specificity of the score at different cutoff points. D, Calibration plot compares the observed (dotted line) vs predicted (solid line) risk of outcome events of unplanned rehospitalizations or death in the first year after hospital discharge. The distribution of outcome over the deciles is indicated by the bar graphs at the bottom, with patients with outcomes represented above the line and those without outcomes represented below the line. AUROC indicates area under the receiver operating characteristic curve.

The prognostic score ranged from 0 to 22 points, with lower scores indicating a lower risk of the outcome. (Table 2). We generated 4 risk strata by grouping patients based on their individual prognostic score (Figure 2A; Table 2). The outcome of unplanned rehospitalization or death in the first year after hospital discharge occurred for 5088 of 16 684 patients (30.5%) with scores between 0 and 4 points and 15 732 of 21 641 patients (72.7%) with 11 points or more (Figure 2B; Table 2). The derivation and validation cohorts had similar distributions of prognostic scores of 0 to 4 points (5088 of 16 684 patients [30.5%] and 471 of 1725 patients [27.3%]) and prognostic scores of 11 points or more (15 732 of 21 641 patients [72.7%] and 5753 of 7952 patients [72.3%]) (Table 2). The discrimination was moderate (AUROC, 0.675; 95% CI, 0.672-0.679) (Figure 2C-D).

### Assessment of Clinical Utility of the Prognostic Score

**Figure 3.**
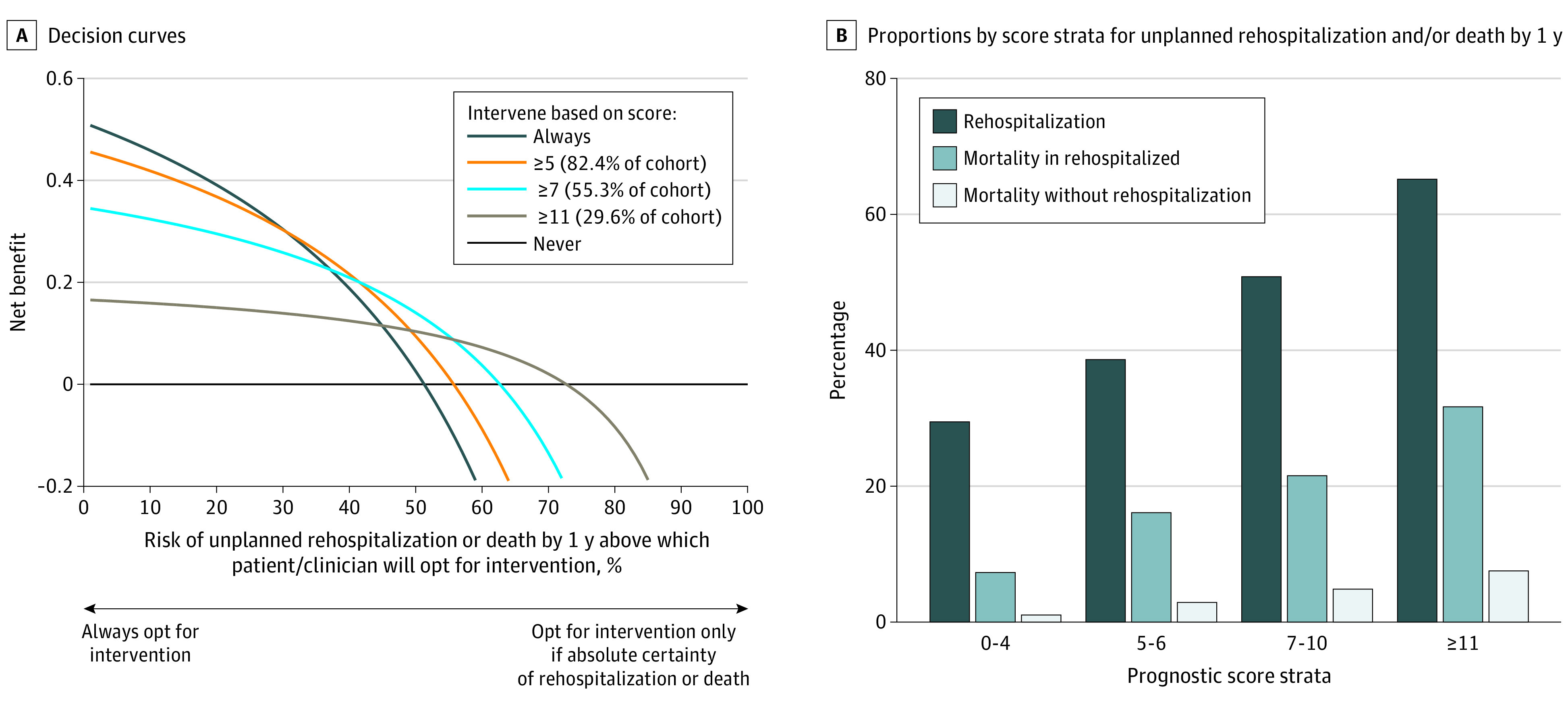
Clinical Usefulness With Decision Curve Analysis (DCA) of Prognostic Score A, The x-axis of DCA graphs refers to the threshold probability, which is the risk of 1-year unplanned rehospitalization or death between 0 and 1, above which the clinician or patient would choose treatment. The y-axis shows the net benefit in units of the benefit associated with correctly identifying 1 unplanned rehospitalization or death. Decision curves are shown for scores of 5 or more, 7 or more, and 11 or more. B, Proportions by score strata for rehospitalization, for deaths in those who experience 1 or more rehospitalization episodes within that strata, and deaths without rehospitalization during the 365-day follow-up period.

Net benefit was maximized with threshold probabilities of 0% to 30% by the treat-all approach. The DCA highlights that the net benefit of this score is positive and higher than the net benefit for the treat-all scenario at threshold probabilities of 30% or more (Figure 3A). In the intervening selective scenario of our analysis, a score of 5 or more has the highest curve for a threshold probability of less than 45%. At a threshold probability of 45% or more and a score of 7 or more, the true positives were 34.7% (32 868 of 94 748 patients), the false positives were 16.6% (15 725 of 94 748 patients), and the estimated net benefit was 0.21; at this cutoff, 55.3% (52 433 of 94 748 patients) of the study cohort may choose follow-up care (eTable 7 in the Supplement and Figure 3A). The individual outcome events mirrored changes in the composite outcome with our prognostic score (Figure 3B and eTable 6 in the Supplement).

### Prognostic Score Validation

The description and performance of our prognostic score in our external validation cohort are shown in eTable 8 and eFigure 2 in the Supplement. The use of predictors during the 200-sample bootstrap validation models is shown in eFigure 3 in the Supplement. With bootstrap internal validation, the mean optimism estimate was 0.0007 (95% CI, 0.0005-0.0010) for the C statistic, −0.0002 (95% CI, −0.0003 to −0.0001) for the Brier score, and 0.0005 (95% CI, 0.0003-0.0006) for the Shapiro R test score.

## Discussion

We derived and validated a parsimonious prognostic score for the outcome of unplanned rehospitalization or death in the first year after hospital discharge, using acute illness characteristics from the index sepsis admission as predictors. The score is calculated using 8 predictors: hospitalizations in the preceding year, age, socioeconomic status using IMD2015 in England quintiles, preadmission dependence, number of comorbidities, admission type, site of infection, and admission blood hemoglobin level, a globally feasible blood test.

What is the clinical value and use of this prognostic score? We and others have highlighted that 1 in 2 sepsis survivors have unplanned rehospitalizations or die in the first year after hospital discharge.^19,29,30,31^ Currently, the follow-up care of sepsis survivors is neither risk based nor uniform within and between health care systems. Seldom does every sepsis survivor receive follow-up care. In this context, we report, to our knowledge, the first prognostic score that helps to identify sepsis survivors’ risk of these 2 patient-centered adverse outcomes. Although predictive accuracy at the individual level is at best modest, our score highlights a clinically useful gradient of risk to target follow-up care. We envision that, in the first instance, the score would be used to inform prognosis discussions with sepsis survivors and follow-up care designed based on patient preferences. More important, because DCA does not mandate information on the costs or effectiveness of treatment or how patients value different health states,^14^ our analysis highlights that it is possible to intervene selectively for sepsis survivors, using a systematic risk-based approach. An example of the follow-up care that we envision would be ensuring better postdischarge adherence to recommended management for common comorbidities, as better adherence could improve sepsis survivor outcomes^32,33^ because effective outpatient management exists for these common rehospitalization diagnoses.^18^ We expect our score to be refined over time based on preliminary use because clinicians and researchers may identify what additional predictors could add value. We expect an increased emphasis on sepsis survivors’ health with unrestricted availability of our score. A similar approach with prognostic scoring for addressing care for older adults after hospitalizations has been reported.^34,35^

The predictors in the final list are consistently reported in the literature as risk factors for rehospitalization^6^ and/or 1-year mortality^7^—hospitalization in the preceding year,^36,37^ increasing age,^19,38,39^ different proxies for socioeconomic status (such as insurance status,^11,38,39,40^ urban residence, and lower income^11^), preadmission dependence, higher comorbidity burden,^10,11,19,38,39,40^ admission type, blood hemoglobin level at admission, and site of infection.^10^ Increased risk of outcome with a lower blood hemoglobin level at index sepsis admission in the score is analogous to the association observed with a lower blood hemoglobin level at critical care discharge.^36,37,38^ We contend that the admission blood hemoglobin level may also represent chronic health status.

### Strengths and Limitations

Our study has some strengths. We present a novel score to stratify sepsis survivors’ risk of rehospitalization or death in the first year after hospital discharge, whereas other tools focus on 30-day rehospitalization risk.^41^ Our primary outcome of all-cause rehospitalizations or death has the lowest risk of ascertainment bias. The score, albeit feasible to measure even in resource-limited settings, requires recalibration and potentially revision before use as standard of care in other health care settings. As part of development work for this analysis, we published the operational definition for sepsis from this database,^3^ long-term mortality in the derivation cohort,^8^ and systematic reviews to identify key predictors.^6,7^ Our method of handling comorbidities functions even when patients have 2 or more comorbidities, is transferable between health care systems that use different comorbidity definitions, and the incremental risk with increasing numbers of comorbidities has clinical validity. We followed the recommendations by Steyerberg and colleagues^27^ for bootstrap validations.

Our study also has some limitations. Our study population was limited to patients who survived to hospital discharge after a critical care unit admission for sepsis. Our model predicts the 1-year risk, not necessarily at specific time points during follow-up. We did not have information on patient preferences after discharge, so 1-year mortality in some cases may reflect a lack of seeking care or limitations of care, such as end-of-life care in community settings.^42,43^ However, this is unlikely to be a major factor because death without rehospitalization was observed for only 4.3% of the derivation cohort (4035 of 94 748) and 4.5% of the validation cohort (1118 of 24 669). Although we used a national deprivation index (IMD2015 in England) as a proxy for socioeconomic strata, there are other national or regional indces. Because additional risk is only for individuals in the lowest socioeconomic status, this is an easy limitation to overcome when using other indices. A similar consideration applies to preadmission dependence assessments. Perhaps the biggest limitation of our score is that currently there are no proven interventions to prevent posthospitalization death or readmissions of sepsis survivors.

Our study highlights several future research questions. Although we report internal and temporal external validations, generalizability of the score should be assessed in other settings, including resource-limited settings. There is insufficient knowledge regarding sepsis survivors’ preferences on the relevant range of threshold probabilities within DCA, which needs to be determined. Because there are no proven interventions to weigh the risk vs benefit within DCA, the utility of the score as a prognostic enrichment^44^ tool could be evaluated by ongoing trials (eg, NCT03565159) and data from completed trials.^45^ The score could be assessed as a benchmarking tool between hospitals to reduce variation in sepsis survivor outcomes.

## Conclusions

In this study, unplanned rehospitalization or death in the first year after hospital discharge were common adverse outcomes among sepsis survivors. This study validated a simple and internationally feasible prognostic score for this composite outcome, which could inform prognosis discussions, trial design, and follow-up care of sepsis survivors.

## Notes

Supplement. **eMethods.** **eFigure 1.** TRIPOD Guideline Informed Structure of the Study Design and Analyses **eFigure 2.** Description of Clinical Prediction Model in Validation Cohort **eFigure 3.** Variable Use During 200 Bootstrap Validation Models **eTable 1.** Risk Factors Reported in Studies **eTable 2.** Individual Comorbidities in Derivation and Validation Cohorts **eTable 3.** Unplanned Readmission Diagnoses, as per the Healthcare Cost and Utilization Project (HCUP) Clinical Classifications Software Multilevel Categories **eTable 4.** Univariate Association of Predictors in Derivation Cohort **eTable 5.** Preliminary Model, Post-Stepwise Reduction Model, and Sequential Models After Dropping Individual Predictors **eTable 6.** Proportion for Deaths, Rehospitalization and Deaths Without Rehospitalization by Score **eTable 7.** Decision Curve Analysis for Score Cutoff ≥7 Points With Threshold Probability of 45% **eTable 8.** Predictors From the Final Model for All Rehospitalization or Death in the First Year Following Hospital Discharge in Sepsis Survivors in the Validation Cohort
