# Supplementary material for: Development, Validation, and Clinical Utility Assessment of a Prognostic Score for 1-Year Unplanned Rehospitalization or Death of Adult Sepsis Survivors
Source: JAMA Netw Open. 2020 Sep 14;3(9):e2013580. doi: 10.1001/jamanetworkopen.2020.13580 (PMC7490647; doi:10.1001/jamanetworkopen.2020.13580)
Supplement: Supplement. — eMethods. eFigure 1. TRIPOD Guideline Informed Structure of the Study Design and Analyses eFigure 2. Description of Clinical Prediction Model in Validation Cohort eFigure 3. Variable Use During 200 Bootstrap Validation Models eTable 1. Risk Factors Reported in Studies eTable 2. Individual Comorbidities in Derivation and Validation Cohorts eTable 3. Unplanned Readmission Diagnoses, as per the Healthcare Cost and Utilization Project (HCUP) Clinical Classifications Software Multilevel Categories eTable 4. Univariate Association of Predictors in Derivation Cohort eTable 5. Preliminary Model, Post-Stepwise Reduction Model, and Sequential Models After Dropping Individual Predictors eTable 6. Proportion for Deaths, Rehospitalization and Deaths Without Rehospitalization by Score eTable 7. Decision Curve Analysis for Score Cutoff ≥7 Points With Threshold Probability of 45% eTable 8. Predictors From the Final Model for All Rehospitalization or Death in the First Year Following Hospital Discharge in Sepsis Survivors in the Validation Cohort [file jamanetwopen-e2013580-s001.pdf]

# Supplementary Online Content

Shankar-Hari M, Rubenfeld GD, Ferrando-Vivas P, Harrison DA, Rowan K. Development, validation, and clinical utility assessment of a prognostic score for 1-year unplanned rehospitalization or death of adult sepsis survivors. *JAMA Netw Open.* 2020;3(9):e2013580. doi:10.1001/jamanetworkopen.2020.13580

**eMethods.**
**eFigure 1.** TRIPOD Guideline Informed Structure of the Study Design and Analyses
**eFigure 2.** Description of Clinical Prediction Model in Validation Cohort
**eFigure 3.** Variable Use During 200 Bootstrap Validation Models
**eTable 1.** Risk Factors Reported in Studies
**eTable 2.** Individual Comorbidities in Derivation and Validation Cohorts
**eTable 3.** Unplanned Readmission Diagnoses, as per the Healthcare Cost and Utilization Project (HCUP) Clinical Classifications Software Multilevel Categories
**eTable 4.** Univariate Association of Predictors in Derivation Cohort
**eTable 5.** Preliminary Model, Post-Stepwise Reduction Model, and Sequential Models After Dropping Individual Predictors
**eTable 6.** Proportion for Deaths, Rehospitalization and Deaths Without Rehospitalization by Score
**eTable 7.** Decision Curve Analysis for Score Cutoff ≥ 7 Points With Threshold Probability of 45%
**eTable 8.** Predictors From the Final Model for All Rehospitalization or Death in the First Year Following Hospital Discharge in Sepsis Survivors in the Validation Cohort

This supplementary material has been provided by the authors to give readers additional information about their work.

© 2020 Shankar-Hari M et al. *JAMA Network Open.*

**eMethods.**

**Study database**

For consecutive critical care admissions, trained data collectors collect sociodemographic, comorbidity, and physiologic data to precise rules and definitions, during the first 24 hours following admission to critical care. Diagnostic data are determined clinically and coded using the five-tier hierarchical ICNARC Coding Method[1]. Collected data undergo extensive local and central validation prior to pooling into the database[2], with patient-level unique identifiers for additional data linkage[1]. Support for the collection and use of these data has been obtained under Section 251 of the National Health Service Act 2006 (PIAG 2–10(f)/2005).

**Sepsis case definition**

We defined the index critical care admission for sepsis as the hospitalization that included first critical care unit admission with a primary or secondary reason for admission to the critical care unit associated with infection and a total Sequential Organ Failure Assessment (SOFA) score from the first 24 hours in the critical care unit ≥ 2 [3].

**Further description of predictors**

Generic predictors were age, sex, ethnicity, the 2015 Index of Multiple Deprivation (IMD2015) in England[4] as proxy for socioeconomic status, preadmission dependence, hospitalizations in the year preceding index sepsis admission, comorbidities, admission type, acute illness severity using APACHE II physiology score[5], hospital length of stay (LOS), worst lactate and hemoglobin concentrations at sepsis critical care admission and type of hospital. Sepsis-specific predictors were site of infection, numbers of organ dysfunction[3,6] and organ support according to National Critical Care Minimum Dataset definitions[7].

IMD2015, refers to the 2015 Index of Multiple Deprivation, the official measure of relative deprivation in England, consisting of income, employment, education, health, crime, barriers to housing services and living environment domains[8]. IMD2015 is a valid proxy for socioeconomic status[9].

The pre-index hospitalization dependency status is a subjective assessment of whether the participant receives no, some or total assistance with daily activities such as bathing, dressing, going to the toilet, moving in/out of bed/chair, continence and eating, prior to ICU admission.

**Critical Care Minimum Dataset organ support definitions**

Advanced respiratory support is indicated by one or more of the following: invasive mechanical ventilatory support applied via a trans-laryngeal tube or tracheostomy; BPAP (bilevel positive airway pressure) applied via a trans-laryngeal tracheal tube or tracheostomy; CPAP (continuous positive airway pressure) via a trans-laryngeal tracheal tube; extracorporeal respiratory support.

Advanced cardiovascular support is indicated by one or more of the following: multiple intravenous and/or rhythm controlling drugs (e.g. inotropes, amiodarone, nitrates etc.), of which at least one must be vasoactive, when used simultaneously to support or control arterial pressure, cardiac output or organ/tissue perfusion; continuous observation of cardiac output and derived indices (e.g. with a

© 2020 Shankar-Hari M et al. *JAMA Network Open.*

pulmonary artery catheter, lithium dilution, pulse contour analyses, esophageal doppler, impedance and conductance methods; an intra-aortic balloon pump in place and other assist devices; temporary cardiac pacemaker (valid each day while connected for therapeutic reasons to a functioning external pacemaker unit). Neurological support is indicated by one or more of the following: central nervous system depression sufficient to prejudice their airway and protective reflexes, except central nervous system depression caused by sedation prescribed to facilitate mechanical ventilation or by poisoning (e.g. deliberate or accidental self-administered overdose, alcohol, drugs etc.); invasive neurological monitoring or treatment (e.g. intracranial pressure, jugular bulb sampling, external ventricular drain etc.); continuous intravenous medication to control seizures and/or for continuous cerebral monitoring; therapeutic hypothermia using cooling protocols or devices. Gastrointestinal support is indicated by the following: parenteral or enteral nutrition (i.e. any method of feeding other than normal oral intake). Renal support is indicated by the following: acute renal replacement therapy (e.g. hemodialysis, hemofiltration).

© 2020 Shankar-Hari M et al. *JAMA Network Open.*

**eFigure 1.** TRIPOD Guideline Informed Structure of the Study Design and Analyses

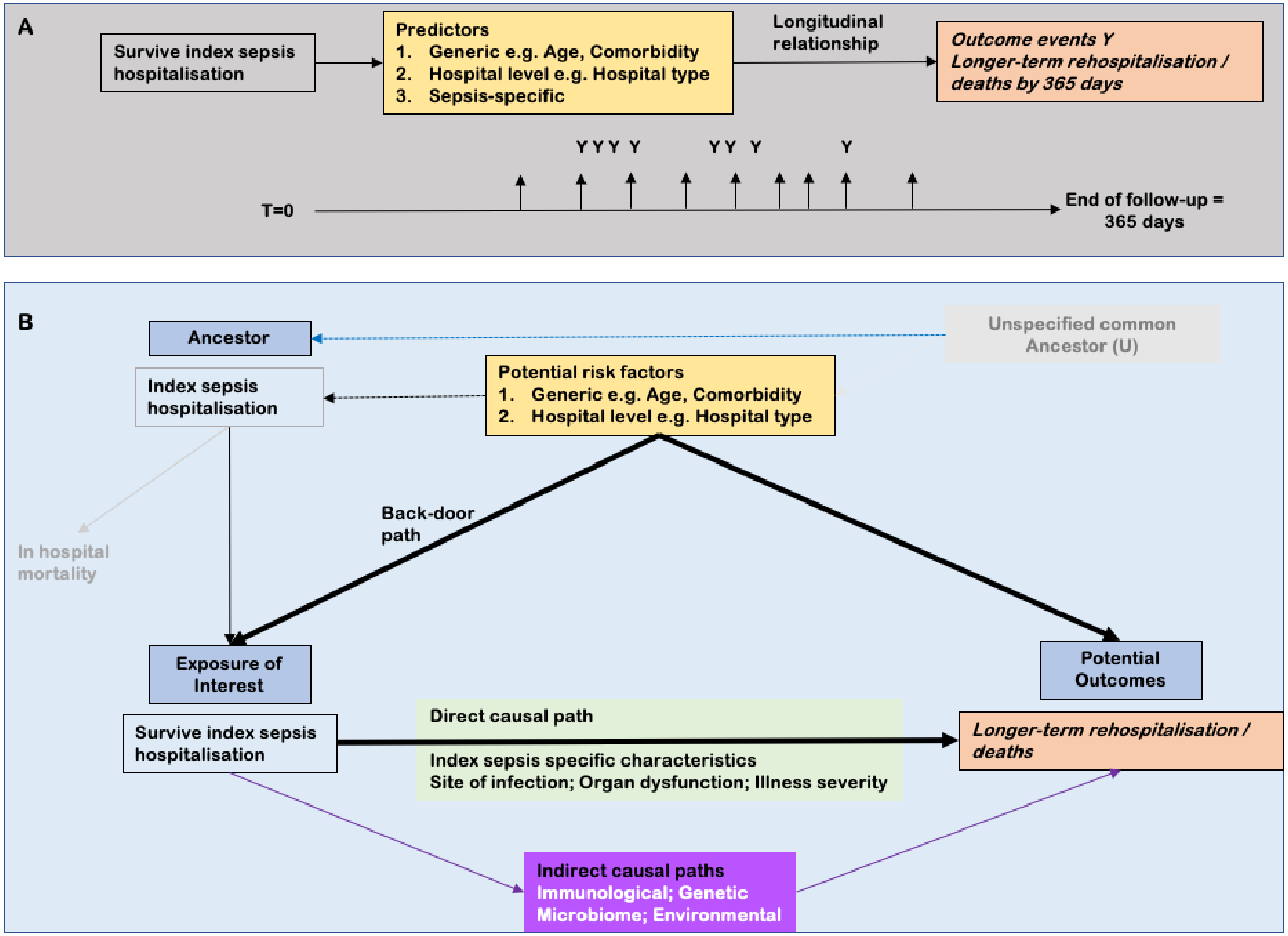


© 2020 Shankar-Hari M et al. *JAMA Network Open*.

**eFigure 2.** Description of Clinical Prediction Model in Validation Cohort

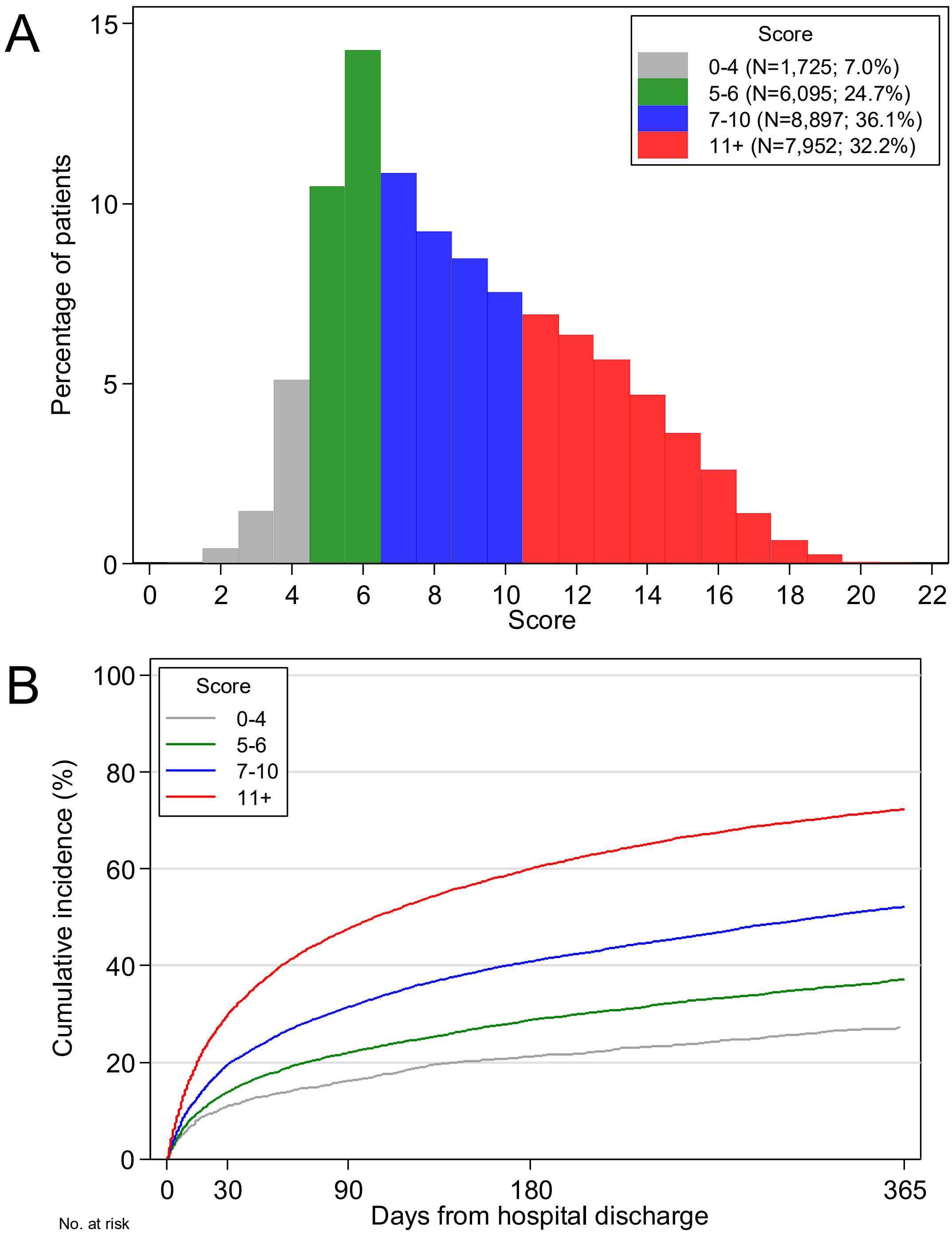


| No. at risk | 0 | 90 | 180 | 365 |
|---|---|---|---|---|
| 0-4 | 1,725 | 1,446 | 1,360 | 1,254 |
| 5-6 | 6,095 | 4,758 | 4,340 | 3,831 |
| 7-10 | 8,897 | 6,104 | 5,266 | 4,262 |
| 11+ | 7,952 | 4,176 | 3,188 | 2,205 |

© 2020 Shankar-Hari M et al. *JAMA Network Open.*

**eFigure 3.** Variable Use During 200 Bootstrap Validation Models

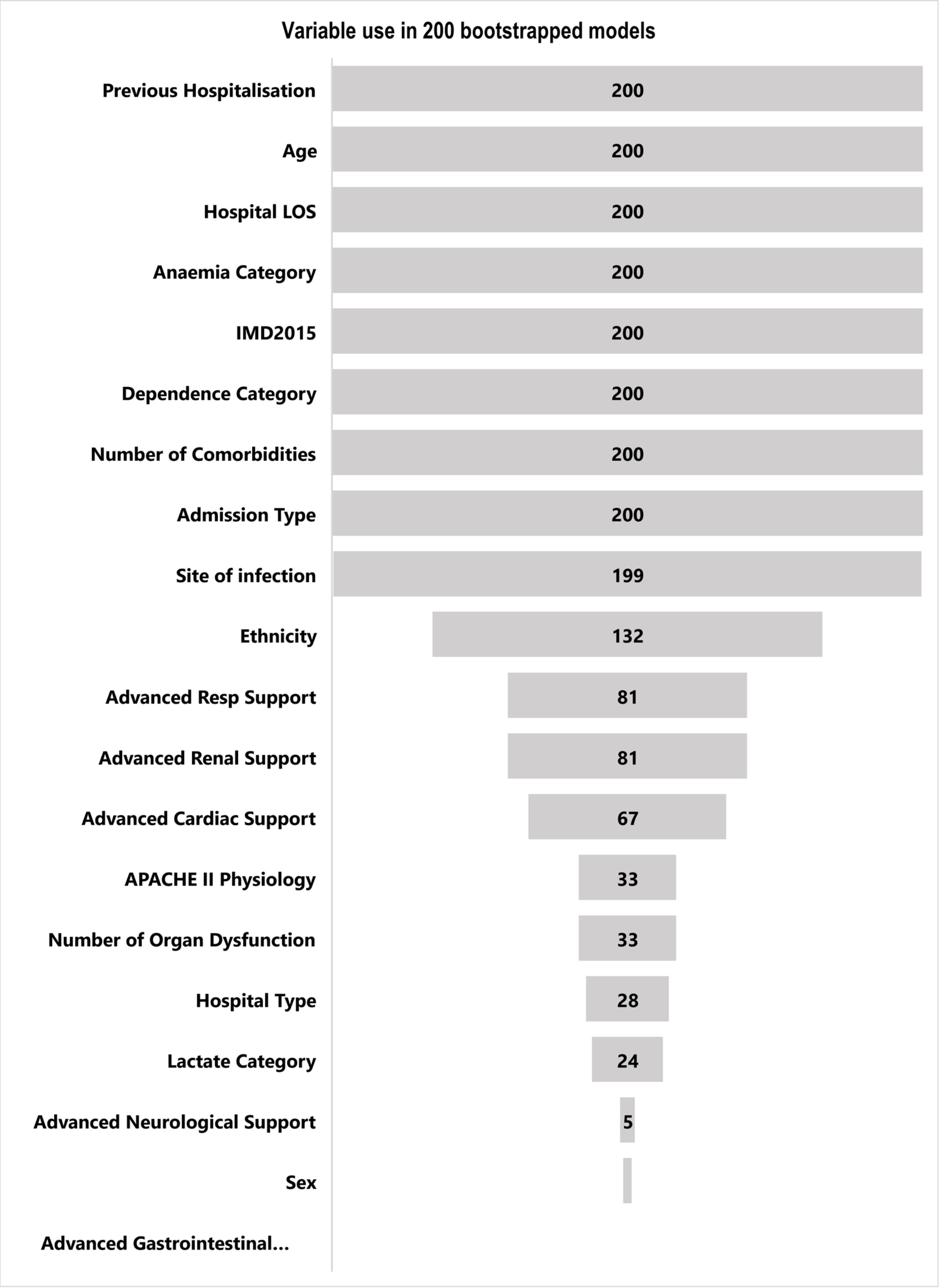


© 2020 Shankar-Hari M et al. *JAMA Network Open.*

**eTable 1.** Risk Factors Reported in Studies

| Reference [ID] | Model and Primary (or rehospitalisation) outcome as reported | Generic characteristics | Sepsis-specific characteristics |
|---|---|---|---|
| Chang DW et al (2015) [10] | Mixed-effects logistic regression for 30-day rehospitalisation | Younger age; Male; Black or Native American; Higher burden of comorbidities; Urban residence; Lower income; Hospitals serving higher proportion of minorities; For profit hospitals; University hospitals | No independent association reported |
| Dietz BW (2016) [11] | Mixed-effects logistic regression for In-hospital death, or transition to hospice during 30-day read-missions | Older age ; Higher burden of comorbidities; Prior hospitalisations; Insurance status; Non-elective index admission; Discharge disposition not to home; Lower discharge levels of haemoglobin; Lower Sodium concentrations; Higher discharge levels of RDW | Sepsis<br>Presence of shock |
| Donnelly JP (2015) [12] | Mixed effects logistic regression for 30-day rehospitalisation | Female; Longer index admission length of stay; Higher burden of comorbidities; Institutions with higher sepsis case volume and lower ICU utilisation | Digestive system infection sites based on ICD-9 codes |
| Goodwin AJ (2015) [13] | Multivariable logistic regression for 30-day rehospitalisation | Age <80years ; Male; Black ; Medicare or Medicaid as primary payer; Comorbidities; Discharge disposition not to home; Institutions with higher sepsis case volume; Higher in-hospital sepsis mortality | Sepsis-specific effect lost significance once comorbidities were accounted |
| Hua M (2015) [14] | Competing risk regression for 30-day rehospitalisation | Older age; Longer index admission length of stay; Higher burden of comorbidities including Dialysis dependent; Medicaid as primary payer; Discharge disposition not to home; Tracheostomy at index admission | Organ dysfunction (described as severe sepsis) |
| Jones TK (2015) [15] | Multivariable logistic regression for 30-day rehospitalisation | Lower age; Hospitalisation in previous year; non-elective index admission; Lower discharge levels of haemoglobin; Higher discharge levels of RDW | No independent association reported |
| Liu V (2014) [16] | Competing risk regression for 30-day rehospitalisation | Older age; Higher burden of comorbidities; Longer index admission length of stay Requirement for ICU care | Illness severity at index-admission |
| Ortego A (2015) [17] | Multivariable logistic regression for 30-day rehospitalisation | Malignancy as comorbidity; Length of stay>4 days; Recent hospitalisation within 30-days | No independent association reported |
| Sun A (2016) [18] | Multivariable logistic regression for 30-day rehospitalisation | Prior hospitalisation before index sepsis episode; Use of Total parenteral nutrition; Longer duration of antibiotics; Lower discharge haemoglobin | No independent association reported |
| Zilberberg MD (2015) [19] | Multivariable logistic regression for 30-day rehospitalisation | No independent association reported | Presence of ESBL<br>Presence of Bacteroides spp<br>Acute Kidney injury |

© 2020 Shankar-Hari M et al. *JAMA Network Open.*

**eTable 2.** Individual Comorbidities in Derivation and Validation Cohorts

| Comorbidities (N; %) | Derivation cohort | Validation cohort |
|---|---|---|
| - Congestive heart failure | 4,480 (4.7%) | 1,421 (5.8%) |
| - Past Myocardial infarction | 1,259 (1.3%) | 1,049 (4.3%) |
| - Peripheral vascular disease | 3,137 (3.3%) | 935 (3.8%) |
| - CVD | 2,031 (2.1%) | 591 (2.4%) |
| - Chronic lung disease | 14,475 (15.3%) | 4,082 (16.6%) |
| - Chronic liver disease | 3,112 (3.3%) | 922 (3.7%) |
| - Chronic kidney disease | 5,561 (5.9%) | 1,656 (6.7%) |
| - Rheumatological disease | 2,087 (2.2%) | 651 (2.6%) |
| - Diabetes Mellitus | 10,093 (10.7%) | 3,057 (12.4%) |
| - Hematological | 3,156 (3.3%) | 899 (3.6%) |
| - Skin conditions | 712 (0.8%) | 226 (0.9%) |
| - Malignancy | 9,049 (9.6%) | 2,437 (9.9%) |
| - Metastatic disease | 2,644 (2.8%) | 798 (3.2%) |
| - HIV / AIDS | 453 (0.5%) | 125 (0.5%) |
| - Immune comorbidity | 6,430 (6.8%) | 1,817 (7.4%) |

© 2020 Shankar-Hari M et al. *JAMA Network Open.*

**eTable 3.** Unplanned Readmission Diagnoses, as per the Healthcare Cost and Utilization Project (HCUP) Clinical Classifications Software Multilevel Categories

| **CCS criteria** | **Within 30-days N (%)** | **Within 90-days N (%)** | **Within 180-days N (%)** | **Within 365-days N (%)** |
|---|---|---|---|---|
| **Infectious** | 3,383 / 16,058 (21.1%) | 5,548 / 26,915 (20.6%) | 7,150 / 34,937 (20.5%) | 9,067 / 44,441 (20.4%) |
| **Circulatory** | 1,770 / 16,058 (11.0%) | 3,009 / 26,915 (11.2%) | 3,935 / 34,937 (11.3%) | 5,072 / 44,441 (11.4%) |
| **Respiratory** | 1,983 / 16,058 (12.4%) | 3,444 / 26,915 (12.8%) | 4,542 / 34,937 (13.0%) | 5,918 / 44,441 (13.3%) |
| **Digestive** | 1,609 / 16,058 (10.0%) | 2,777 / 26,915 (10.3%) | 3,583/ 34,937 (10.3%) | 4,627 / 44,441 (10.4%) |
| **Genitourinary** | 797 / 16,058 (5.0%) | 1.303 / 26,915 (4.8%) | 1,693 / 34,937 (4.9%) | 2,102 / 44,441 (4.7%) |
| **Endocrine, Metabolic, Nutrition and Immunity** | 639 / 16,058 (4.0%) | 1,091 / 26,915 (4.1%) | 1,369 / 34,937 (3.9%) | 1,660 / 44,441 (3.7%) |
| **Neoplastic** | 623 / 16,058 (3.9%) | 1,126 / 26,915 (4.2%) | 1,484 / 34,937 (4.3%) | 1,850 / 44,441 (4.2%) |
| **Dermatologic and Musculoskeletal** | 514 / 16,058 (3.2%) | 941 / 26,915 (3.5%) | 1,240 / 34,937 (3.6%) | 1,660 / 44,441 (3.7%) |
| **Hematologic** | 229 / 16,058 (1.4%) | 424 / 26,915 (1.6%) | 556 / 34,937 (1.6%) | 656 / 44,441 (1.5%) |
| **Nervous system** | 451 / 16,058 (2.8%) | 860 / 26,915 (3.2%) | 1,204 / 34,937 (3.5%) | 1,564 / 44,441 (3.5%) |
| **Injury and Poisoning** | 2.431 / 16,058 (15.1%) | 3,743 / 26,915 (13.9%) | 4,716 / 34,937 (13.5%) | 5,784 / 44,441 (13.3%) |
| **All others** | 1,629 / 16,058 (10.1%) | 2,649 / 26,915 (9.8%) | 3.465 / 34,937 (9.9%) | 4,404 / 44,441 (9.9%) |
| **Total** | 16,058 / 94,748 (16.9%) | 26,915 / 94,748 (28.4%) | 34,937 / 94,748 (36.9%) | 44,441 / 94,748 (46.9%) |

© 2020 Shankar-Hari M et al. *JAMA Network Open.*

**eTable 4.** Univariate Association of Predictors in Derivation Cohort

Detailed characteristics of derivation cohort (n=94,748) are presented in Table-1 main manuscript.

The number of primary outcome events were 48,594 (51.3%).

| Parameter | Odds ratio (95% CI) | *P* Value |
|---|---|---|
| **Age category (years)** | | |
| - **<30** | 1 | - |
| - **30 - 39** | 1.11 (1.04 – 120) | 0.004 |
| - **40 - 49** | 1.44 (1.35 – 1.54) | <0.001 |
| - **50 - 59** | 1.54 (1.45 – 1.64) | <0.001 |
| - **60 - 69** | 1.69 (1.59 – 1.79) | <0.001 |
| - **70 - 79** | 1.86 (1.75 – 1.97) | <0.001 |
| - **≥ 80 years** | 2.14 (2.01 – 2.28) | <0.001 |
| **Sex** | | |
| - **Females** | 1 | - |
| - **Male** | 1.04 (1.02 – 1.07) | 0.001 |
| **Ethnicity (N; %)** | | |
| - **White** | 1 | - |
| - **Asian** | 0.94 (0.88 – 1.01) | 0.085 |
| - **Black** | 0.88 (0.80 – 0.96) | 0.003 |
| - **Other** | 0.75 (0.70 – 0.81) | <0.001 |
| **English IMD quintiles (N; %)** | | |
| - **IMD-1 (Least)** | 1 | - |
| - **IMD-2** | 1.06 (1.01 – 1.11) | 0.01 |
| - **IMD-3** | 1.08 (1.03 – 1.13) | 0.001 |
| - **IMD-4** | 1.10 (1.06 – 1.15) | <0.001 |
| - **IMD-5 (Most)** | 1.21 (1.16 – 1.26) | <0.001 |
| **Pre-admission dependence (N; %)** | | |
| - **None** | 1 | - |
| - **Moderate** | 1.99 (1.93 – 2.05) | <0.001 |
| - **ADL** | 2.68 (2.37 – 3.04) | <0.001 |
| **Comorbidities (Base category = No)** | | |
| - **Congestive heart failure** | 2.72 (2.54 – 2.91) | <0.001 |
| - **Past Myocardial infarction** | 2.49 (2.20 – 2.82) | <0.001 |
| - **Peripheral vascular disease** | 2.13 (1.97 – 2.30) | <0.001 |
| - **CVD** | 2.34 (2.13 – 2.58) | <0.001 |
| - **Chronic lung disease** | 2.23 (2.15 – 2.32) | <0.001 |
| - **Chronic liver disease** | 2.20 (1.88 – 2.18) | <0.001 |
| - **Chronic kidney disease** | 2.90 (2.73 – 3.09) | <0.001 |
| - **Rheumatological disease** | 2.02 (1.84 – 2.21) | <0.001 |
| - **Diabetes Mellitus** | 2.17 (2.07 – 2.27) | <0.001 |
| - **Haematological** | 2.73 (2.52 – 2.96) | <0.001 |
| - **Skin conditions** | 2.96 (2.49 – 3.51) | <0.001 |
| - **Malignancy** | 2.07 (1.98 – 2.17) | <0.001 |
| - **Metastatic disease** | 3.13 (2.86 – 3.43) | <0.001 |
| - **Immune comorbidity** | 2.26 (2.14 – 2.38) | <0.001 |
| **Hospitalization in preceding year** | | |
| - **0** | 1 | |
| - **1** | 1.52 (1.47 – 1.57) | <0.001 |
| - **2** | 2.08 (1.99 – 2.16) | <0.001 |
| - **3 or more** | 3.58 (3.45 – 3.71) | <0.001 |

© 2020 Shankar-Hari M et al. *JAMA Network Open.*

| | | |
|---|---|---|
| **Admission type** | | |
| - **Medical** | 1 | - |
| - **Elective surgical** | 0.79 (0.75 – 0.83) | <0.001 |
| - **Emergency surgical** | 0.82 (0.80 – 0.85) | <0.001 |
| **Index illness severity per 5-point increase** | | |
| - **APACHE II physiology score** | 1.12 (1.11 – 1.14) | <0.001 |
| **Anaemia on admission day (Haemoglobin)** | | |
| - **>11** | 1 | - |
| - **9.1 – 11** | 1.93 (1.80 – 2.06) | <0.001 |
| - **7.1 – 9** | 1.16 (1.56 – 1.67) | <0.001 |
| - **<=7** | 1.25 (1.21 – 1.29) | <0.001 |
| **Site of infection** | | |
| - **Respiratory** | 1 | - |
| - **Cardiovascular** | 1.27 (1.15 – 1.41) | <0.001 |
| - **Gastrointestinal** | 0.90 (0.87 – 0.92) | <0.001 |
| - **Genitourinary** | 1.03 (0.98 – 1.09) | 0.21 |
| - **Musculoskeletal/Dermatological** | 0.92 (0.87 – 0.97) | 0.004 |
| - **Neurological** | 0.55 (0.51 – 0.59) | <0.001 |
| - **Unknown** | 1.23 (1.16 – 1.30) | <0.001 |
| **Numbers of organ dysfunction** | | |
| - **I** | 1 | - |
| - **2** | 1.15 (1.10 – 1.21) | <0.001 |
| - **3** | 1.25 (1.20 – 1.31) | <0.001 |
| - **4** | 1.33 (1.21 – 1.38) | <0.001 |
| - **5** | 1.29 (1.21 – 1.38) | <0.001 |
| **Advanced organ support (base category =** | | |
| **No)** | 0.87 (0.85 – 0.89) | <0.001 |
| - **Respiratory** | 0.98 (0.95 – 1.01) | 0.26 |
| - **Cardiovascular** | 1.22 (1.18 – 1.28) | <0.001 |
| - **Renal** | 1.03 (1.10 – 1.06) | 0.008 |
| - **Gastrointestinal** | 0.96 (0.91 – 1.01) | 0.16 |
| - **Neurological** | | |
| **Hospital length of stay at index admission per 7 days** | 1.05 (1.04 – 1.105) | <0.001 |

© 2020 Shankar-Hari M et al. *JAMA Network Open.*

**eTable 5.** Preliminary Model, Post-Stepwise Reduction Model, and Sequential Models After Dropping Individual Predictors

| Covariate | PRELIMINARY MODEL | POST STEP-WISE REDUCTION model for p<0.001 | Complex predictor | Predictors with lowest coefficients | | | FINAL MODEL |
|---|---|---|---|---|---|---|---|
| | | | AP2APSdropped | Ethnicity dropped | CVS support dropped | Hosp LOS dropped | Hospital type dropped |
| Previous hospitalisation | | | | | | | |
| - 0 | 1 | 1 | 1 | 1 | 1 | 1 | 1 |
| - 1 | 1.21 (1.16 – 2.26) | 1.21 (1.17 – 1.26) | 1.21 (1.16 – 1.25) | 1.21 (1.17 – 1.26) | 1.21 (1.17 – 1.26) | 1.22 (1.18 – 1.27) | 1.22 (1.18 – 1.27) |
| - 2 | 1.46 (1.39 – 1.53) | 1.45 (1.39 – 1.52) | 1.45 (1.38 – 1.52) | 1.45 (1.39 – 1.52) | 1.46 (1.39 – 1.53) | 1.47 (1.40 – 1.54) | 1.47 (1.40 – 1.54) |
| - 3 or more | 2.17 (2.06 – 2.28) | 2.17 (2.07 – 2.27) | 2.16 (2.06 – 2.26) | 2.17 (2.07 – 2.27) | 2.17 (2.07 – 2.27) | 2.18 (2.09 – 2.29) | 2.19 (2.09 – 2.30) |
| Age categories | | | | | | | |
| - <30 years | 1 | 1 | 1 | 1 | 1 | 1 | 1 |
| - 30 – 39 | 1.08 (0.99 – 1.18) | 1.07 (0.99 – 1.16) | 1.07 (0.99 – 1.16) | 1.07 (0.99 – 1.16) | 1.07 (0.99 – 1.16) | 1.10 (1.01 – 1.18) | 1.10 (1.01 – 1.18) |
| - 40 – 49 | 1.30 (1.20 – 1.40) | 1.30 (1.21 – 1.39) | 1.30 (1.21 – 1.39) | 1.31 (1.22 – 1.40) | 1.30 (1.21 – 1.40) | 1.35 (1.26 – 1.44) | 1.35 (1.26 – 1.44) |
| - 50 – 59 | 1.30 (1.21 – 1.40) | 1.29 (1.21 – 1.38) | 1.29 (1.21 – 1.38) | 1.31 (1.22 – 1.40) | 1.30 (1.22 – 1.39) | 1.36 (1.27 – 1.45) | 1.36 (1.27 – 1.45) |
| - 60 – 69 | 1.35 (1.25 – 1.45) | 1.35 (1.27 – 1.44) | 1.35 (1.27 – 1.44) | 1.38 (1.29 – 1.47) | 1.37 (1.28 – 1.46) | 1.44 (1.35 – 1.54) | 1.44 (1.35 – 1.54) |
| - 70 – 79 | 1.46 (1.36 – 1.57) | 1.49 (1.40 – 1.59) | 1.49 (1.40 – 1.59) | 1.51 (1.42 – 1.61) | 1.51 (1.41 – 1.61) | 1.59 (1.49 – 1.70) | 1.59 (1.49 – 1.69) |
| - 80 year or older | 1.80 (1.66 – 1.95) | 1.83 (1.70 – 1.96) | 1.83 (1.70 – 1.96) | 1.86 (1.73 – 1.99) | 1.85 (1.73 – 1.99) | 1.94 (1.81 – 2.08) | 1.94 (1.81 – 2.07) |
| Male (vs female) | 1.03 (1.00 – 1.06) | Dropped | | | | | |
| Ethnicity (vs white) | | | | | | | |
| - White | 1 | 1 | 1 | | | | |
| - Asian | 0.89 (0.82 – 0.97) | 0.90 (0.84 – 0.97) | 0.90 (0.84 – 0.97) | Dropped | | | |
| - Black | 0.82 (0.74 – 0.92) | 0.84 (0.77 – 0.93) | 0.84 (0.77 – 0.93) | | | | |
| - Other | 0.85 (0.78 – 0.92) | 0.86 (0.80 – 0.93) | 0.86 (0.80 – 0.93) | | | | |
| IMD 2015 | | | | | | | |
| - 1 (least deprived) | 1 | 1 | 1 | 1 | 1 | 1 | 1 |
| - 2 | 1.06 (1.01 – 1.11) | 1.05 (1.00 – 1.10) | 1.05 (1.00 – 1.10) | 1.05 (1.00 – 1.10) | 1.05 (1.01 – 1.10) | 1.05 (1.00 – 1.10) | 1.05 (1.00 – 1.10) |
| - 3 | 1.08 (1.03 – 1.14) | 1.08 (1.03 – 1.13) | 1.08 (1.03 – 1.13) | 1.08 (1.03 – 1.13) | 1.08 (1.03 – 1.13) | 1.07 (1.03 – 1.12) | 1.07 (1.02 – 1.12) |
| - 4 | 1.12 (1.06 -1.17) | 1.12 (1.07 – 1.17) | 1.12 (1.07 – 1.17) | 1.11 (1.06 – 1.16) | 1.11 (1.06 – 1.16) | 1.10 (1.06 – 1.15) | 1.10 (1.06 – 1.16) |
| - 5 (most deprived) | 1.24 (1.18 – 1.30) | 1.24 (1.19 – 1.30) | 1.24 (1.19 – 1.30) | 1.23 (1.18 – 1.29) | 1.23 (1.18 – 1.29) | 1.23 (1.18 – 1.28) | 1.23 (1.18 – 1.29) |
| Dependency (vs none) | | | | | | | |
| - None | 1 | 1 | 1 | 1 | 1 | 1 | 1 |
| - Some (minor or major) | 1.48 (1.43 – 1.53) | 1.50 (1.45 – 1.55) | 1.51 (1.46 – 1.56) | 1.51 (1.46 – 1.56) | 1.51 (1.46 – 1.56) | 1.54 (1.49 – 1.59) | 1.54 (1.49 – 1.59) |
| - Total | 2.57 (2.20 – 3.00) | 2.61 (2.28 – 2.99) | 2.64 (2.31 – 3.01) | 2.65 (2.32 – 3.03) | 2.66 (2.33 – 3.04) | 2.80 (2.45 – 3.20) | 2.80 (2.45 – 3.20) |
| Comorbidities (vs none) | | | | | | | |
| - 0 | 1 | 1 | 1 | 1 | 1 | 1 | 1 |
| - 1 | 1.37 (1.31 -1.43) | 1.40 (1.34 – 1.45) | 1.40 (1.34 – 1.45) | 1.40 (1.34 – 1.45) | 1.40 (1.34 – 1.45) | 1.38 (1.33 – 1.44) | 1.39 (1.33 – 1.44) |
| - 2 | 1.71 (1.62 – 1.81) | 1.75 (1.66 – 1.85) | 1.75 (1.66 – 1.85) | 1.75 (1.66 – 1.85) | 1.75 (1.66 – 1.85) | 1.72 (1.63 – 1.82) | 1.72 (1.63 – 1.82) |
| - 3 | 2.11 (1.95 – 2.30) | 2.12 (1.95 – 2.30) | 2.23 (2.06 – 2.41) | 2.22 (2.06 – 2.41) | 2.23 (2.06 – 2.41) | 2.17 (2.01 – 2.35) | 2.18 (2.02 – 2.36) |
| - 4 or more | 2.73 (2.38 – 3.13) | 2.73 (2.38 – 3.13) | 2.84 (2.49 – 3.23) | 2.84 (2.49 – 3.23) | 2.84 (2.49 – 3.23) | 2.74 (2.41 – 3.12) | 2.74 (2.41 – 3.12) |
| Admission type | | | | | | | |
| - Elective surgical | 1 | 1 | 1 | 1 | 1 | 1 | 1 |
| - Emergency surgical | 1.26 (1.1.9 – 1.34) | 1.26 (1.1.9 – 1.34) | 1.26 (1.18 – 1.35) | 1.27 (1.19 – 1.35) | 1.26 (1.18 – 1.34) | 1.29 (1.21 – 1.37) | 1.29 (1.21 – 1.37) |
| - Medical | 1.43 (134 – 1.52) | 1.43 (134 – 1.52) | 1.45 (1.36 – 1.55) | 1.45 (1.36 – 1.55) | 1.44 (1.36 – 1.54) | 1.48 (1.39 – 1.58) | 1.48 (1.39 – 1.57) |
| AP2APS 5-point increments | 1.02 (1.00 – 1.04) | 1.03 (1.01 – 1.04) | Dropped | | | | |
| Hospital LOS 7-day increments | 1.04 (1.02 – 1.10) | 1.04 (1.03 – 1.04) | 1.04 (1.03 – 1.04) | 1.04 (1.03 – 1.04) | 1.04 (1.03 – 1.04) | Dropped | |
| Admission highest lactate value | | Dropped | | | | | |

© 2020 Shankar-Hari M et al. *JAMA Network Open.*

| | | | | | | | |
|---|---|---|---|---|---|---|---|
| - <2mmol/L<br>- 2mmol/L – 4mmol/L<br>- >4mmol/L | 1<br>1.01 (0.97 – 1.06)<br>1.06 (1.00 – 112) | | | | | | |
| Admission haemoglobin<br>- >11<br>- 9 – 11<br>- 7 – 9<br>- <7 | <br>1<br>1.06 (1.02 – 1.10)<br>1.17 (1.12 – 1.22)<br>1.27 (1.17 – 1.37) | <br>1<br>1.06 (1.03 – 1.10)<br>1.17 (1.13 – 1.22)<br>1.30 (1.21 – 1.39) | <br>1<br>1.07 (1.04 – 1.11)<br>1.19 (1.15 – 1.23)<br>1.33 (1.24 – 1.43) | <br>1<br>1.07 (1.03 – 1.10)<br>1.19 (1.14 – 1.23)<br>1.32 (1.23 – 1.41) | <br>1<br>1.07 (1.03 – 1.10)<br>1.19 (1.14 – 1.23)<br>1.32 (1.22 – 1.41) | <br>1<br>1.10 (1.07 – 1.14)<br>1.27 (1.23 – 1.32)<br>1.45 (1.35 – 1.55) | <br>1<br>1.10 (1.07 – 1.14)<br>1.28 (1.23 – 1.33)<br>1.46 (1.36 – 1.56) |
| Site of infection<br>- Neurological<br>- Cardiovascular<br>- Gastrointestinal<br>- Genitourinary<br>- Musculoskeletal/Dermatological<br>- Respiratory<br>- Unknown | <br>1<br>1.43 (1.23 – 1.65)<br>1.32 (1.20 – 1.45)<br>1.27 (1.14 – 1.41)<br>1.12 (1.00 – 1.25)<br>1.35 (1.23 – 1.48)<br>1.34 (1.20 – 1.50) | <br>1<br>1.43 (1.25 – 1.63)<br>1.36 (1.25 – 1.48)<br>1.30 (1.19 – 1.43)<br>1.18 (1.07 – 1.30)<br>1.36 (1.26 – 1.78)<br>1.40 (1.27 – 1.54) | <br>1<br>1.42 (1.25 – 1.62)<br>1.35 (1.24 – 1.47)<br>1.31 (1.19 – 1.43)<br>1.18 (1.07 – 1.30)<br>1.36 (1.26 – 1.47)<br>1.41 (1.28 – 1.55) | <br>1<br>1.44 (1.26 – 1.64)<br>1.36 (1.25 – 1.48)<br>1.31 (1.20 – 1.44)<br>1.19 (1.08 – 1.31)<br>1.37 (1.26 – 1.48)<br>1.41 (1.28 – 1.55) | <br>1<br>1.42 (1.25 – 1.62)<br>1.35 (1.24 – 1.47)<br>1.30 (1.19 – 1.43)<br>1.18 (1.07 – 1.30)<br>1.36 (1.26 – 1.47)<br>1.39 (1.27 – 1.53) | <br>1<br>1.45 (1.27 – 1.65)<br>1.30 (1.19 – 1.41)<br>1.19 (1.08 – 1.30)<br>1.18 (1.06 – 1.29)<br>1.30 (1.20 – 1.40)<br>1.33 (1.21 – 1.47) | <br>1<br>1.45 (1.27 – 1.65)<br>1.30 (1.19 – 1.41)<br>1.19 (1.08 – 1.30)<br>1.17 (1.06 – 1.29)<br>1.30 (1.21 – 1.46)<br>1.33 (1.21 – 1.46) |
| Number of organ dysfunction<br>- 1<br>- 2<br>- 3<br>- 4<br>- 5 or more | <br>1<br>1.06 (1.00 – 1.12)<br>1.08 (1.02 – 1.14)<br>1.10 (1.04 – 1.17)<br>1.05 (0.96 – 1.14) | Dropped | | | | | |
| Advanced Respiratory support | 0.92 (0.89 – 0.96) | Dropped | | | | | |
| Advanced Cardiovascular support | 0.93 (0.89 – 0.96) | 0.93 (0.90 – 0.96) | 0.94 (0.91 – 0.97) | 0.94 (0.91 – 0.97) | Dropped | | |
| Advanced Renal support | 1.01 (0.95 – 1.06) | Dropped | | | | | |
| Advanced Gastrointestinal support | 1.02 (0.98 – 1.05) | Dropped | | | | | |
| Advanced Neurological support | 1.06 (1.00 – 1.14) | Dropped | | | | | |
| Hospital type<br>- Non-University<br>- University | <br>1<br>1.05 (1.02 – 1.09) | <br>1<br>1.05 (1.02 – 1.08) | <br>1<br>1.05 (1.02 – 1.08) | <br>1<br>1.04 (1.01 – 1.08) | <br>1<br>1.04 (1.01 – 1.06) | <br>1<br>1.06 (1.03 – 1.09) | Dropped |
| AUROC (95% CI)<br><br>Brier score | 0.681 (0.677 – 0.685)<br>0.23 | 0.683 (0.680 – 0.687)<br>0.22 | 0.683 (0.680 – 0.686)<br>0.23 | 0.683 (0.680 – 0.687)<br>0.22 | 0.683 (0.679 – 0.686)<br>0.23 | 0.675 (0.672 – 0.679)<br>0.23 | 0.675 (0.672 – 0.679)<br>0.23 |

© 2020 Shankar-Hari M et al. *JAMA Network Open.*

**eTable 6.** Proportion for Deaths, Rehospitalization and Deaths Without Rehospitalization by Score

| Event | Score category | Derivation cohort | Validation cohort |
|---|---|---|---|
| Death | 0-4 | 531 / 16684 (3.2%) | 32 / 1,725 (1.9%) |
| | 5-6 | 2,333 / 25,631 (9.1%) | 362 / 6,095 (5.9%) |
| | 7-11 | 4,860 / 30,791 (15.8%) | 1,192 / 8,897 (13.4%) |
| | ≥ 11 | 6,095 / 21,642 (28.2%) | 2,126 / 7,952 (26.7%) |
| Rehospitalization | 0-4 | 4,915 / 16684 (29.5%) | 461 / 1,725 (26.7%) |
| | 5-6 | 9,897 / 25,631 (38.6%) | 2,142 / 6,095 (35.1%) |
| | 7-11 | 15,645 / 30,791 (50.8%) | 4,229 / 8,897 (47.5%) |
| | ≥ 11 | 14,102 / 21,642 (65.2%) | 5,179 / 7,952 (65.1%) |
| Death without rehospitalisation | 0-4 | 173 / 16684 (1.0%) | 10 / 1,725 (0.6%) |
| | 5-6 | 740 / 25,631 (2.9%) | 125 / 6,095 (2.1%) |
| | 7-11 | 1,492 / 30,791 (4.9%) | 409 / 8,897 (4.6%) |
| | ≥ 11 | 1,630 / 21,642 (7.5%) | 574 / 7,952 (7.2%) |

© 2020 Shankar-Hari M et al. *JAMA Network Open.*

**eTable 7.** Decision Curve Analysis for Score Cutoff ≥ 7 Points With Threshold Probability of 45%

| **score ≥ 7** | **Outcome event** | | **Total** |
|---|---|---|---|
| | **Yes** | **No** | |
| **Yes** | 32,868 (True Positive) | 19,565 (False Negative) | 52,433 |
| **No** | 15,725 (False Positive) | 26,590 (True Negative) | 42,315 |
| **Total** | 48,594 | 46,154 | 94,748 (Total N) |

© 2020 Shankar-Hari M et al. *JAMA Network Open.*

**eTable 8.** Predictors From the Final Model for All Rehospitalization or Death in the First Year Following Hospital Discharge in Sepsis Survivors in the Validation Cohort

| Risk factor known at index sepsis admission | Validation cohort | |
|---|---|---|
| | N with outcome / N at risk (%) | Adjusted odds ratio (95% CI) |
| Previous hospitalization | | |
| - 0 | 4,259 / 10,429 (40.8%) | 1 |
| - 1 | 2,752 / 5,272 (52.2%) | 1.25 (1.15 – 1.34) |
| - 2 | 1,867 / 3,082 (60.6%) | 1.54 (1.40 – 1.70) |
| - 3 or more | 4,251 / 5,886 (72.2%) | 2.15 (1.96 – 2.36) |
| Age in 10-year increments | | |
| - <30 | 539 /1,281 (42.1%) | 1 |
| - 30 – 39 | 688 / 1,529 (45.0%) | 1.17 (0.99 – 1.37) |
| - 40 – 49 | 1,308 / 2,706 (51.0%) | 1.25 (1.08 – 1.45) |
| - 50 – 59 | 2,006 /3,934 (51.0%) | 1.30 (1.13 – 1.49) |
| - 60 – 69 | 3,077 / 5,793 (53.1%) | 1.37 (1.20 – 1.57) |
| - 70- 79 | 3,341 / 5,851 (57.1%) | 1.60 (1.40 – 1.84) |
| - 80+ | 2,170 / 3,575 (60.7%) | 1.94 (1.68 – 2.24) |
| IMD2015 quintile | | |
| - 1 (least deprived) | 1,917 / 3,737 (51.3%) | 1 |
| - 2 | 2,179 / 4,156 (52.4%) | 1.02 (0.92 – 1.12) |
| - 3 | 2,525 / 4,732 (53.4%) | 1.10 (1.00 – 1.20) |
| - 4 | 2,696 / 5,086 (53.0%) | 1.09 (1.00 – 1.19) |
| - 5 (most deprived) | 3,3238 / 6006 (55.4%) | 1.21 (1.11 – 1.32) |
| Pre-admission dependence | | |
| - None | 8,703 / 17,925 (48.6%) | 1 |
| - Moderate | 4,132 / 6,300 (65.6%) | 1.52 (1.43 – 1.63) |
| - All | 249 / 342 (72.8%) | 2.88 (2.22 – 3.75) |
| Co-morbidity | | |
| - 0 | 6,089 / 14,232 (42.8%) | 1 |
| - 1 | 3,121 / 5,131 (60.8%) | 1.40 (1.30 – 1.52) |
| - 2 | 2,193 / 3,148 (69.7%) | 1.73 (1.56 – 1.91) |
| - 3 | 1,106 / 1,438 (76.9%) | 2.23 (1.93 – 2.58) |
| - 4+ | 620 / 720 (86.1%) | 3.76 (2.97 – 4.75) |
| Admission | | |
| - Elective surgical | 628 / 1, 345 (46.7%) | 1 |
| - Emergency surgical | 2,877 / 5,943 (48.4%) | 1.44 (1.26 – 1.64) |
| - Medical | 9,623 / 17,379 (55.4%) | 1.76 (1.55 – 2.00) |
| Haemoglobin at admission | | |
| - >11 | 4,518 / 9,540 (47.4%) | 1 |
| - 9.1 – 11 | 4,564 / 8,594 (53.1%) | 1.06 (0.99 – 1.13) |
| - 7.1 – 9 | 3,357 / 5,464 (61.4%) | 1.30 (1.21 – 1.40) |
| - <=7 | 563 / 824 (68.3%) | 1.68 (1.43 – 1.99) |
| Site of infection | | |
| - Neurological | 334 / 781 (42.8%) | 1 |
| - Respiratory | 6,117 / 11,348 (53.9%) | 1.12 (0.95 – 1.31) |
| - Cardiovascular | 229 / 391 (58.6%) | 1.42 (1.08 – 1.85) |
| - Gastrointestinal | 3,624 / 7,210 (50.3%) | 1.16 (0.96 – 1.35) |
| - Genitourinary | 1,103 / 1,960 (56.3%) | 1.08 (0.90 – 1.29) |
| - Musculoskeletal/Derm | 791 / 1,430 (55.3%) | 1.23 (1.01 – 1.49) |
| - Unknown | 930 / 1,549 (60.0%) | 1.26 (1.04 – 1.52) |
| | | |
| SSIP score category | N with outcome / N at risk (%) | Adjusted odds ratio (95% CI) |
| 0 – 4 points (low risk) | 471 / 1,725 (27.3%) | 1 |
| 5 – 6 points | 2,267 / 6,095 (37.2%) | 1.58 (1.40 – 1.77) |
| 7 – 10 points | 4,638 / 8,897 (52.1%) | 2.90 (2.59 – 3.25) |
| >=11 points (high risk) | 5,753 / 7,952 (72.4%) | 6.97 (6.20 – 7.83) |
| Totals | 13,129 / 24,669 (53.2%) | |

**eReferences**

© 2020 Shankar-Hari M et al. *JAMA Network Open.*

1. Young JD, Goldfrad C, Rowan K. Development and testing of a hierarchical method to code the reason for admission to intensive care units: the ICNARC Coding Method. Intensive Care National Audit & Research Centre. Br J Anaesth. 2001;87(4):543-548.
2. Harrison DA, Brady AR, Rowan K. Case mix, outcome and length of stay for admissions to adult, general critical care units in England, Wales and Northern Ireland: the Intensive Care National Audit & Research Centre Case Mix Programme Database. Crit Care. 2004;8(2):R99-111.
3. Shankar-Hari M, Harrison DA, Rubenfeld GD, Rowan K. Epidemiology of sepsis and septic shock in critical care units: comparison between sepsis-2 and sepsis-3 populations using a national critical care database. Br J Anaesth. 2017;119(4):626-636.
4. https://www.gov.uk/government/statistics/english-indices-of-deprivation-2015
5. Quach S, Hennessy DA, Faris P, Fong A, Quan H, Doig C. A comparison between the APACHE II and Charlson Index Score for predicting hospital mortality in critically ill patients. BMC Health Serv Res. 2009;9:129.
6. Singer M, Deutschman CS, Seymour CW, et al. The Third International Consensus Definitions for Sepsis and Septic Shock (Sepsis-3). JAMA. 2016;315(8):801-810.
7. Information Standards Board for H, Social C. Critical Care Minimum Dataset Full Specification Critical Care Minimum Dataset Version 8.0. 2010.
8. https://www.gov.uk/government/statistics/english-indices-of-deprivation-2015
9. https://www.ifs.org.uk/comms/r79.pdf
10. Chang DW, Tseng CH, Shapiro MF. Rehospitalizations Following Sepsis: Common and Costly. Crit Care Med. 2015;43(10):2085-2093.
11. Dietz BW, Jones TK, Small DS, Gaieski DF, Mikkelsen ME. The Relationship Between Index Hospitalizations, Sepsis, and Death or Transition to Hospice Care During 30-Day Hospital Readmissions. Medical care. 2016.
12. Donnelly JP, Hohmann SF, Wang HE. Unplanned Readmissions After Hospitalization for Severe Sepsis at Academic Medical Center-Affiliated Hospitals. Crit Care Med. 2015;43(9):1916-1927.
13. Goodwin AJ, Rice DA, Simpson KN, Ford DW. Frequency, cost, and risk factors of readmissions among severe sepsis survivors. Crit Care Med. 2015;43(4):738-746.
14. Hua M, Gong MN, Brady J, Wunsch H. Early and late unplanned rehospitalizations for survivors of critical illness*. Crit Care Med. 2015;43(2):430-438.
15. Jones TK, Fuchs BD, Small DS, et al. Post-Acute Care Use and Hospital Readmission after Sepsis. Annals of the American Thoracic Society. 2015;12(6):904-913.
16. Liu V, Lei X, Prescott HC, Kipnis P, Iwashyna TJ, Escobar GJ. Hospital readmission and healthcare utilization following sepsis in community settings. J Hosp Med. 2014;9(8):502-507.
17. Ortego A, Gaieski DF, Fuchs BD, et al. Hospital-based acute care use in survivors of septic shock. Crit Care Med. 2015;43(4):729-737.
18. Sun A, Netzer G, Small DS, et al. Association Between Index Hospitalization and Hospital Readmission in Sepsis Survivors. Crit Care Med. 2016;44(3):478-487.
19. Zilberberg MD, Shorr AF, Micek ST, Kollef MH. Risk factors for 30-day readmission among patients with culture-positive severe sepsis and septic shock: A retrospective cohort study. J Hosp Med. 2015;10(10):678-685.

© 2020 Shankar-Hari M et al. *JAMA Network Open.*
